# Prognostic Factors and Predictors of In-Hospital Mortality Among COVID-19 Patients Admitted to the Intensive Care Unit: An Aid for Triage, Counseling, and Resource Allocation

**DOI:** 10.7759/cureus.16577

**Published:** 2021-07-23

**Authors:** Waleed Burhamah, Iman Qahi, Melinda Oroszlányová, Sameera Shuaibi, Razan Alhunaidi, May Alduwailah, Maryam Alhenaidi, Zahraa Mohammad

**Affiliations:** 1 School of Medicine, Royal College of Surgeons in Ireland, Dublin, IRL; 2 Department of General Surgery, Mubarak AlKabeer Hospital, Kuwait, KWT; 3 College of Engineering and Technology, American University of the Middle East, Kuwait, KWT; 4 Department of Internal Medicine, Al-Adan Hospital, Kuwait, KWT; 5 Department of Internal Medicine, Kuwait University, Health Sciences Center, School of Medicine, Kuwait, KWT

**Keywords:** covid-19, icu, critical care, sars-cov- 2, prognostic scores

## Abstract

Background: The severe acute respiratory syndrome coronavirus 2 (SARS-CoV-2) remains today a global health pandemic. Those with severe infection are at risk of rapid clinical deterioration; as a result, intensive care unit (ICU) admission is not uncommon in such patients. A number of determinants have been identified as predictors of poor prognosis and in-hospital mortality, ranging from demographic characteristics, laboratory and/or radiological findings.

Aim: To identify determinants of in-hospital mortality and examine the accuracy of seven early warning scores in predicting in-hospital mortality.

Methods: This is a retrospective study conducted in Kuwait from July 2020 to March 2021, and participants were adult patients with a positive test on the real-time polymerase chain reaction (RT-PCR) for SARS-CoV-2 and who met the criteria for ICU admission. Data collected included: demographics, clinical status on hospital arrival, laboratory test results, and ICU course. Furthermore, we calculated seven early warning scores for each of our patients.

Results: A total of 133 patients were admitted to our COVID-19 ICU with a median age of 59 years. Arrival to ICU on mechanical ventilation (MV), developing in-hospital complications, having chronic kidney disease (CKD), having a high white blood count (WBC), lactate dehydrogenase (LDH), lactate, or urea levels were found to be significant predictors of in-hospital mortality. Furthermore, the 4C mortality score for COVID-19, VACO index for COVID-19 mortality, and the PRIEST COVID-19 clinical severity score proved to be the most superior in predicting in-hospital mortality.

Conclusion: Identifying high-risk patients and those with a poor prognosis allows for efficient triaging and the delivery of high-standard care while minimizing the strain on the healthcare system.

## Introduction

In December 2019, a cluster of cases of pneumonia of unknown etiology was reported in the City of Wuhan, China. Shortly, the novel severe acute respiratory syndrome coronavirus 2 (SARS-CoV- 2) was identified as the culprit. It remains today a global health pandemic responsible for more than 3 million deaths worldwide [1]. 

This virus is a newly identified strain that belongs to a family of viruses known as the Coronaviruses. Seven are known to cause disease in humans, including the culprits behind the outbreaks of severe acute respiratory syndrome (SARS) and middle east respiratory syndrome (MERS) [2]. The mode of human-to-human transmission is mainly through respiratory secretions [3]. Various incubation periods have been reported by numerous studies, a period of 2 to 14 days seems to be the general consensus [4,5]. Clinically, it manifests as a syndrome with diverse symptomatology and a spectrum of severities, ranging from being asymptomatic or merely a flu-like illness to as devastatingly as respiratory failure, septic shock, multiple organ dysfunction, and death [6,7]. Of the COVID-19 patients requiring hospital admission, it is estimated that 25% also meet the requirements for intensive care unit (ICU) admission [8,9].

A number of determinants have been identified as indicators of severe infection and poor prognosis. Patients with severe COVID-19 infection are a group of high-risk patients, they initially manifest with a clinical picture of high respiratory rate (usually ≥30 breaths per minute), oxygen saturation of ≤93% and lung infiltrates >50% on chest X-ray [10]. Other indicators of a severe infection include high fever (>39 °C), lymphopenia, elevated liver and renal function tests (aspartate aminotransferase, alanine aminotransferase, creatinine, and urea), elevated acute-phase reactants (C-reactive protein (CRP), procalcitonin (PCT), and serum ferritin), and elevated coagulation profile parameters (prothrombin time, fibrinogen, and D-dimer) [11]. This subset of patients are at high risk for rapid clinical deterioration and the development of respiratory failure [9] Furthermore, a number of early warning scores have been generated and validated for use in COVID-19 patients [12-18]; they each differ in clinical variables but ultimately aid in the prediction of patient outcomes and in-hospital mortality.

During a pandemic involving a novel pathogen with unknown virulence and that presents with a spectrum of severities, a strain on the healthcare system and resources available is inevitable. As a result, risk stratification and efficient triaging are crucial; this allows adequate resource allocation in order to deliver a high standard of care that is patient-centered. Furthermore, it aids in the counseling of the patient’s relatives to relay a more realistic picture regarding the outcomes of the patient.

On February 24, 2019, the Kuwait News Agency (KUNA) reported the first few cases of the SARS-CoV- 2 virus in Kuwait, identified in a number of travelers arriving from abroad. Despite the efforts to limit the spread of this virus, the number of cases escalated and as of today has amounted to 294,694 cases and 1703 mortalities [19]. This is a single-center study conducted in Kuwait. In this study, we aim to describe the characteristics of the patients admitted to our COVID-19 intensive care unit (ICU). Second, we aim to identify the determinants of in-hospital mortality. Lastly, we examine and compare the accuracy of a number of early warning scores, investigating their performance in our cohort.

## Materials and methods

Study design and participants

This is a retrospective observational study conducted at a university-affiliated hospital in Kuwait, during the period of July 2020 to March 2021. At our hospital, we allocated two of our internal medicine wards and one of our ICUs to the care of COVID-19 patients. Our dedicated COVID-19 ICU is composed of 25 beds and is run by a team of certified ICU staff, internal medicine residents, and nursing staff that provide one-to-one patient care around the clock. Study participants were adult patients (above 18 years of age) with a positive test on the real-time polymerase chain reaction (RT-PCR) for the SARS-CoV-2 virus, and who met the criteria for ICU admission either from the emergency department (ED) or the COVID-19 wards. Ethical approval was obtained from the ethical committee in the Ministry of Health, Kuwait.

Data collection

We reviewed the clinical records of our ICU patients. The following data were collected: demographics (age, gender, and nationality), comorbidities, smoking history, and the use of angiotensin-converting enzyme inhibitors (ACEI). We recorded the patient's vital signs and symptoms upon hospital arrival as well as the duration of symptoms (days). Furthermore, details regarding the ICU course were recorded including, the source of ICU admission (ward vs. ED), the mode of ventilation on ICU arrival (MV vs. NIV), laboratory test results on day 1 of ICU, need for mechanical ventilation (MV) during ICU stay, duration of MV (days), length of ICU stay (days), and treatments received. Our primary outcome was in-hospital mortality. 

Early warning scores

For each of our patients, we calculated seven early warning scores that have been previously validated. The Modified Early Warning Score (MEWS) for Clinical Deterioration [12], the National Early Warning Score 2 (NEWS2) [13], the Quick COVID-19 Severity Index (qCSI) [14], the International Severe Acute Respiratory Infection Consortium Clinical Characterization Protocol (4C mortality score for COVID-19) [15], the Veterans Health Administration COVID-19 (VACO) Index for COVID-19 Mortality [16], The MuLBSTA Score for Viral Pneumonia Mortality (MulBTSA) [17], and the Pandemic Respiratory Infection Emergency System Triage Severity Score (PRIEST) [18].

Statistical analysis

The analysis of our data was performed using R software, version 4.0.5 (R Foundation for Statistical Computing, Vienna, Austria). Continuous variables were reported as median (interquartile range - IQR), and categorical variables were reported as frequency (percentage). In order to compare the clinical records of patients who were discharged and those who died, the continuous variables were tested by the t-test and Mann-Whitney test (at α = 0.05), and the categorical variables were tested by using the Chi-squared test (at α = 0.05). To determine potential risk factors for in-hospital death, the odds ratios (OR) were calculated with the 95% confidence intervals (CI) and the corresponding p-values. Furthermore, the Area Under the Receiver Operating Characteristics (AUROC) metric was calculated for each mortality score to evaluate its performance in predicting in-hospital mortality. Youden’s index was used to estimate optimal cut-off points and corresponding sensitivity and specificity.

## Results

A total of 133 patients were admitted to our COVID-19 ICU with a median age of 59 years and a median length of stay of seven days. The majority (65%) were transferred from the COVID-19 wards after deteriorating and only 35% were directly admitted to ICU from the ED. Most of the patients were males (68%), non-Kuwaiti (68%), and non-smokers (90%). Less than half of the patients (41%) survived, and 59% died in the hospital (Table 1). The majority of our cohort had medical comorbidities (84%), with the most common being diabetes mellitus (57%) followed by hypertension (55%; Table 2). In addition, the most common symptoms on hospital admission were shortness of breath (73%), fever (68%), and cough (60%), with a median duration of four days (Table 3). Vital signs on hospital arrival are summarized in Table 4 and the day 1 laboratory results are summarized in Table 5.

**Table 1:** Clinical characteristics of the patients 7 n (%); median (IQR).

	N = 133^7^
Nationality	
Kuwaiti	42 (32%)
Non-Kuwaiti	91 (68%)
Age	59 (49, 68)
Gender	
Female	43 (32%)
Male	90 (68%)
Smoker	
No	120 (90%)
Yes	13 (9.8%)
Direct admission to ICU	
No (ward first)	87 (65%)
Yes (from ED to ICU)	46 (35%)
Length of ICU stay (days)	7 (3, 13)
Outcome	
Death	78 (59%)
Discharged	55 (41%)

**Table 2:** Co-morbidities 7 n (%); median (IQR). ACEI: angiotensin-converting enzyme inhibitor, COPD: chronic obstructive pulmonary disease, CKD: chronic kidney disease, OSA: obstructive sleep apnea.

Comorbidities	N=133
Previously healthy	
No	112 (84%)
Yes	21 (16%)
Diabetes mellitus	
Yes	76 (57%)
Hypertension	
Yes	73 (55%)
Taking an ACEI	
Yes	36 (27%)
Dyslipidemia	
Yes	30 (23%)
Ischemic heart disease	
Yes	31 (23%)
Asthma	
Yes	13 (9.8%)
COPD	
Yes	4 (3.0%)
CKD	
Yes	23 (17%)
Malignancy	
Yes	5 (3.8%)
OSA	
Yes	13 (9.8%)
Rheumatologic condition	
Yes	3 (2.3%)
Hypothyroidism	
Yes	12 (9.0%)

**Table 3:** Symptoms on hospital admission ^7^n (%); median (IQR).

Symptoms on hospital admission	N = 1 33^7^
Duration of symptoms (days)	4.0 (1.0, 6.0)
Cough	80 (60%)
Fever	90 (68%)
Shortness of breath	97 (73%)
Headache	3 (2.3%)
Chest pain	8 (6.0%)
Arthralgia/myalgia	6 (4.5%)
Vomiting	16 (12%)
Diarrhea	13 (9.8%)
Fatigue	31 (23%)
Altered consciousness	12 (9.0%)

**Table 4:** Vital signs on hospital arrival 7n (%); median (IQR). HR: heart rate, SBP: systolic blood pressure, DBP: diastolic blood pressure, Sat on RA: saturation on room air, RR: diastolic blood pressure.

Vitals on hospital arrival	N = 133^7^
HR	102 (89, 119)
Unknown	11
SBP	128(116, 148)
Unknown	10
DBP	74 (66, 82)
Unknown	10
Oxygen Sat on RA	88 (82, 90)
Unknown	12
Temperature	37.90 (37.20, 38.70)
Unknown	14
RR	30 (22, 38)
Unknown	55

**Table 5:** Laboratory results on day 1 ^7^n (%); median (IQR). Hb: hemoglobin, WBC: white blood count, CRP: C-reactive protein, PCT: procalcitonin, LDH: lactate dehydrogenase, ALT: alanine transaminase, AST: aspartate aminotransferase.

Investigations	N = 133^7^
Hb (g/L)	126 (108, 138)
WBC (10^9^/L)	11 (8, 16)
Lymphocyte count (10^9^/L)	0.80 (0.50, 1.40)
Platelet count (10^9^/L)	250 (179, 345)
CRP (mg/L)	118 (70, 211)
Unknown	17
PCT (ng/mL)	1 (0, 2)
Unknown	12
D-dimer (ng/mL)	791 (447, 3,070)
Unknown	13
LDH (IU/L)	622 (432, 832)
Unknown	23
Ferritin (ng/L)	773 (382, 1,500)
Unknown	26
Lactate (mmol/L)	2.12 (1.37, 3.05)
Unknown	33
Creatinine (µmol/L)	89 (72, 136)
Urea (mmol/L)	12 (8, 18)
ALT (IU/L)	40 (26, 72)
AST (IU/L)	58 (37, 88)
Albumin (g/L)	31.0 (26.0, 34.0)
Troponin (ng/L)	35 (13, 190)
Unknown	6

Upon arrival to the ICU, 47% of the patients were already on MV but eventually, 74% of the ICU patients required MV (Table 6). In terms of management, most of the patients received antibiotics (95%), 20% received Oseltamivir, and only 5.3% received both Oseltamivir and Kaletra. Additionally, half of our patients received Dexamethasone (50%; Table 7). The majority of our cohort developed in-hospital complications (78%), with the most common being acute kidney injury (AKI; 46%), coagulopathy (14%), and upper gastrointestinal bleeding (8.3%). Almost half of the patients developed septic shock (47%; Table 8). 

**Table 6:** Ventilation MV: mechanical ventilation, NIV: non-invasive ventilation.

Ventilation	
Arrival at ICU on	
MV	62(47%)
NIV	71(53%)
Eventually required MV	
No	35(26%)
Yes	98(74%)
Intubated on ICU on day	1.00(1-3)
Duration of MV (days)	5.00(3-10.75)
Tracheostomy	
No	128(96%)
Yes	5(3.8%)
Extubated successfully	
No	75(56%)
Was never intubated	35(26%)
Yes	23(17%

**Table 7:** Treatments ^7^n (%); median (IQR). IVIG: intravenous immunoglobulin, NO: nitric oxide.

Treatments	N = 1 33^7^
Antibiotics	
No	6 (4.5%)
Yes	127 (95%)
Antiviral	
No	100 (75%)
Oseltamivir	26 (20%)
Oseltamivir and Lopinavir/Ritonavir	7 (5.3%)
Plasma exchange	
No	113 (85%)
Yes	20 (15%)
Tocilizumab	
No	114 (86%)
Yes	19 (14%)
Dexamethasone	
No	67 (50%)
Yes	66 (50%)
Anakinra	
No	127 (95%)
Yes	6 (4.5%)
IVIG	
No	130 (98%)
Yes	3 (2.3%)
NO	
No	115 (86%)
Yes	18 (14%)
Proning	
No	80 (60%)
Yes	53 (40%)

**Table 8:** Complications ^7^n (%); median (IQR). AKI: acute kidney injury, ROSC: return of spontaneous circulation, DIC: disseminated intravascular coagulation, LL: lower limb, DVT: deep vein thrombosis, PE: pulmonary embolism, MI: myocardial infarction, CVA: cerebrovascular accident, UGI: upper gastrointestinal bleeding, DKA: diabetic ketoacidosis.

Complications	N = 133^7^
In-hospital complications	
No	29 (22%)
Yes	104 (78%)
In-hospital AKI	61 (46%)
In-hospital cardiac arrest with ROSC	10 (7.5%)
In-hospital coagulopathy	18(14%)
Types of coagulopathies	
DIC	10 (7.5%)
DIC and LL ischemia	2 (1.5%)
DIC, LL ischemia, bowel ischemia, and renal ischemia	1 (0.8%)
DVT	1 (0.8%)
LL ischemia	1 (0.8%)
No	115 (86%)
PE	3 (2.3%)
In-hospital MI	8 (6.0%)
In-hospital CVA	4 (3.0%)
In-hospital pneumothorax	8 (6.0%)
In-hospital UGI	11 (8.3%)
In-hospital rhabdomyolysis	7 (5.3%)
In-hospital DKA	4 (3.0%)
In-hospital infective endocarditis	4 (3.0%)
Shock	
Cardiogenic shock	5 (3.8%)
No	66 (50%)
Septic shock	62 (47%)

Table 9 shows the comparison in the clinical characteristics between the two groups of patients; those who died in the hospital and those who were discharged. The average age was almost ten years higher for the patients who died (62.6 years) and the majority of deaths were males. The symptoms and their average duration were similar. Most of the vital signs on hospital arrival were on average similar between the two groups. The average white blood cell count (WBC), lactate dehydrogenase (LDH), lactate, and urea levels were significantly higher in patients who died than those who were discharged.

**Table 9:** Comparison of clinical characteristics and mortality *Mean (SD). ICU: intensive care unit, ED: emergency department, ACEI: angiotensin-converting enzyme inhibitor, COPD: chronic obstructive pulmonary disease, CKD: chronic kidney disease, OSA: obstructive sleep apnea, HR: heart rate, SBP: systolic blood pressure, DBP: diastolic blood pressure, RA: room air, RR: diastolic blood pressure, Hb: hemoglobin, WBC: white blood count, CRP: C-reactive protein, PCT: procalcitonin, LDH: lactate dehydrogenase, ALT: alanine transaminase, AST: aspartate aminotransferase,MV: mechanical ventilator, IVIG: intravenous immunoglobulin, ROSC: return of spontaneous circulation, AKI: acute kidney injury, PE: pulmonary embolism, DIC: disseminated intravascular coagulation, LL: lower limb, DVT: deep vein thrombosis, MI: myocardial infarction, CVA: cerebrovascular accident, UGI: upper gastrointestinal, DKA: diabetic ketoacidosis, MEWS: Modified Early Warning Score for Clinical Deterioration, NEWS2: National Early Warning Score 2, qCSI: Quick COVID-19 Severity Index, 4C score: International Severe Acute Respiratory Infection Consortium Clinical Characterization Protocol (4C mortality score for COVID-19), VACO Index: Veterans Health Administration COVID-19 Index for COVID-19 Mortality, MuLBSTA: MuLBSTA Score for Viral Pneumonia Mortality, PRIEST score: Pandemic Respiratory Infection Emergency System Triage Severity Score.

Characteristics	Death (N=78)	Discharged (N=55)	p-Value
Nationality			0.236
Kuwaiti	21 (26.9%)	21 (38.2%)	
Non-Kuwaiti	57 (73.1%)	34 (61.8%)	
Age*	62.6 (13.7)	53.2 (14.5)	<0.001
Gender			0.787
Female	24 (30.8%)	19 (34.5%)	
Male	54 (69.2%)	36 (65.5%)	
Smoker			0.505
No	72 (92.3%)	48 (87.3%)	
Yes	6 (7.69%)	7 (12.7%)	
Direct admission to ICU			0.016
No (ward first)	58 (74.4%)	29 (52.7%)	
Yes (from ED to ICU)	20 (25.6%)	26 (47.3%)	
Length of ICU stay (days)*	8.83 (7.29)	10.7 (10.1)	0.239
Previously healthy			0.567
No	64 (82.1%)	48 (87.3%)	
Yes	14 (17.9%)	7 (12.7%)	
Comorbidities	Death (N=78)	Discharged (N=55)	p-Value
DM			0.741
No	32 (41.0%)	25 (45.5%)	
Yes	46 (59.0%)	30 (54.5%)	
Hypertension			0.55
No	33 (42.3%)	27 (49.1%)	
Yes	45 (57.7%)	28 (50.9%)	
Taking ACEI			0.026
No	63 (80.8%)	34 (61.8%)	
Yes	15 (19.2%)	21 (38.2%)	
Dyslipidemia			0.703
No	59 (75.6%)	44 (80.0%)	
Yes	19 (24.4%)	11 (20.0%)	
Ischemic heart disease			0.167
No	56 (71.8%)	46 (83.6%)	
Yes	22 (28.2%)	9 (16.4%)	
Asthma			0.266
No	68 (87.2%)	52 (94.5%)	
Yes	10 (12.8%)	3 (5.45%)	
COPD			0.642
No	75 (96.2%)	54 (98.2%)	
Yes	3 (3.85%)	1 (1.82%)	
CKD			0.02
No	59 (75.6%)	51 (92.7%)	
Yes	19 (24.4%)	4 (7.27%)	
Malignancy			1
No	75 (96.2%)	53 (96.4%)	
Yes	3 (3.85%)	2 (3.64%)	
OSA			1
No	70 (89.7%)	50 (90.9%)	
Yes	8 (10.3%)	5 (9.09%)	
Rheumatologic condition			0.267
No	75 (96.2%)	55 (100%)	
Yes	3 (3.85%)	0 (0.00%)	
Hypothyroidism			0.552
No	72 (92.3%)	49 (89.1%)	
Yes	6 (7.69%)	6 (10.9%)	
Symptoms on hospital admission	Death (N=78)	Discharged (N=55)	p-Value
Duration of symptoms (days)*	4.69 (4.69)	3.98 (3.21)	0.302
Cough			0.569
No	29 (37.2%)	24 (43.6%)	
Yes	49 (62.8%)	31 (56.4%)	
Fever			0.915
No	26 (33.3%)	17 (30.9%)	
Yes	52 (66.7%)	38 (69.1%)	
Shortness of breath			1
No	21 (26.9%)	15 (27.3%)	
Yes	57 (73.1%)	40 (72.7%)	
Headache			0.569
No	77 (98.7%)	53 (96.4%)	
Yes	1 (1.28%)	2 (3.64%)	
Chest pain			0.065
No	76 (97.4%)	49 (89.1%)	
Yes	2 (2.56%)	6 (10.9%)	
Arthralgia/myalgia			1
No	74 (94.9%)	53 (96.4%)	
Yes	4 (5.13%)	2 (3.64%)	
Vomiting			0.95
No	68 (87.2%)	49 (89.1%)	
Yes	10 (12.8%)	6 (10.9%)	
Diarrhea			0.064
No	74 (94.9%)	46 (83.6%)	
Yes	4 (5.13%)	9 (16.4%)	
Fatigue			0.894
No	59 (75.6%)	43 (78.2%)	
Yes	19 (24.4%)	12 (21.8%)	
Altered consciousness			0.76
No	70 (89.7%)	51 (92.7%)	
Yes	8 (10.3%)	4 (7.27%)	
Vitals on hospital arrival	Death (N=78)	Discharged (N=55)	p-Value
HR*	105 (21.2)	105 (22.6)	0.945
SBP*	128 (27.7)	136 (24.2)	0.131
DBP*	71.8 (14.4)	78.4 (12.7)	0.009
Sats on RA*	84.1 (12.7)	83.7 (12.4)	0.864
Temp*	38.0 (0.95)	37.9 (1.03)	0.884
RR*	30.6 (9.40)	29.8 (10.1)	0.732
Labs on Day 1 in ICU	Death (N=78)	Discharged (N=55)	p-Value
Hb*	122 (32.9)	121 (29.1)	0.839
WBC*	15.1 (11.8)	10.9 (5.35)	0.007
Lymphocyte count*	1.16 (1.50)	1.27 (1.08)	0.618
Platelet count*	277 (157)	281 (135)	0.884
CRP*	162 (139)	322 (1228)	0.383
PCT*	10.6 (31.9)	8.06 (24.2)	0.617
D-Dimer*	2485 (3761)	2621 (5694)	0.882
LDH*	745 (348)	515 (209)	<0.001
Ferritin*	1895 (3177)	2363 (8652)	0.726
Lactate*	3.92 (3.82)	2.20 (1.77)	0.003
Creatinine*	3578 (29771)	111 (171)	0.307
Urea*	18.3 (13.5)	11.7 (10.0)	0.002
ALT*	132 (466)	52.4 (48.0)	0.139
AST*	223 (715)	68.8 (72.6)	0.063
Albumin*	29.1 (6.48)	31.9 (4.63)	0.004
Troponin*	589 (1727)	1637 (6817)	0.274
Ventilation	Death	Discharged	p-value
Arrival at ICU on			<0.001
MV	54 (69.2%)	8 (14.5%)	
NIV	24 (30.8%)	47 (85.5%)	
Eventually required MV			<0.001
No	0 (0.00%)	35 (63.6%)	
Yes	78 (100%)	20 (36.4%)	
Intubated on ICU day	2.50 (3.02)	0.96 (1.82)	<0.001
Duration of MV (days)*	7.03 (6.52)	3.51 (6.59)	0.003
Tracheostomy			1
No	75 (96.2%)	53 (96.4%)	
Yes	3 (3.85%)	2 (3.64%)	
Extubated successfully			<0.001
No	74 (94.9%)	1 (1.82%)	
Was never intubated	0 (0.00%)	35 (63.6%)	
Yes	4 (5.13%)	19 (34.5%)	
Treatments	Death (N=78)	Discharged (N=55)	p-value
Antibiotics			0.081
No	1 (1.28%)	5 (9.09%)	
Yes	77 (98.7%)	50 (90.9%)	
Antiviral			0.807
No	57 (73.1%)	43 (78.2%)	
Oseltamivir	16 (20.5%)	10 (18.2%)	
Oseltamivir and Lopinavir/Ritonavir	5 (6.41%)	2 (3.64%)	
Plasma exchange			0.063
No	62 (79.5%)	51 (92.7%)	
Yes	16 (20.5%)	4 (7.27%)	
Tocilizumab			0.091
No	63 (80.8%)	51 (92.7%)	
Yes	15 (19.2%)	4 (7.27%)	
Dexamethasone			0.029
No	46 (59.0%)	21 (38.2%)	
Yes	32 (41.0%)	34 (61.8%)	
Anakinra			0.691
No	75 (96.2%)	52 (94.5%)	
Yes	3 (3.85%)	3 (5.45%)	
IVIG			1
No	76 (97.4%)	54 (98.2%)	
Yes	2 (2.56%)	1 (1.82%)	
Nitric oxide			<0.001
No	60 (76.9%)	55 (100%)	
Yes	18 (23.1%)	0 (0.00%)	
Proning			0.112
No	42 (53.8%)	38 (69.1%)	
Yes	36 (46.2%)	17 (30.9%)	
Complications	Death (N=78)	Discharged (N=55)	p-Value
In-hospital complications			<0.001
No	4 (5.13%)	25 (45.5%)	
Yes	74 (94.9%)	30 (54.5%)	
In-hospital AKI			<0.001
No	30 (38.5%)	42 (76.4%)	
Yes	48 (61.5%)	13 (23.6%)	
In-hospital cardiac arrest with ROSC			0.005
No	68 (87.2%)	55 (100%)	
Yes	10 (12.8%)	0 (0.00%)	
In-hospital coagulopathy			0.627
No	66 (84.6%)	49 (89.1%)	
Yes	12 (15.4%)	6 (10.9%)	
Types of coagulopathies			0.019
DIC	9 (11.5%)	1 (1.82%)	
DIC and LL ischemia	1 (1.28%)	1 (1.82%)	
DIC, LL ischemi, bowel ischemia and renal ischemia	1 (1.28%)	0 (0.00%)	
DVT	0 (0.00%)	1 (1.82%)	
LL ischemia	1 (1.28%)	0 (0.00%)	
No	66 (84.6%)	49 (89.1%)	
PE	0 (0.00%)	3 (5.45%)	
In-hospital MI			1
No	73 (93.6%)	52 (94.5%)	
Yes	5 (6.41%)	3 (5.45%)	
In-hospital CVA			0.027
No	78 (100%)	51 (92.7%)	
Yes	0 (0.00%)	4 (7.27%)	
In-hospital pneumothorax			0.469
No	72 (92.3%)	53 (96.4%)	
Yes	6 (7.69%)	2 (3.64%)	
In-hospital UGI bleed			0.122
No	69 (88.5%)	53 (96.4%)	
Yes	9 (11.5%)	2 (3.64%)	
In-hospital rhabdomyolysis			0.447
No	75 (96.2%)	51 (92.7%)	
Yes	3 (3.85%)	4 (7.27%)	
In-hospital DKA			1
No	76 (97.4%)	53 (96.4%)	
Yes	2 (2.56%)	2 (3.64%)	
In-hospital infective endocarditis			1
No	76 (97.4%)	53 (96.4%)	
Yes	2 (2.56%)	2 (3.64%)	
Shock			<0.001
Cardiogenic shock	5 (6.41%)	0 (0.00%)	
No	19 (24.4%)	47 (85.5%)	
Septic shock	54 (69.2%)	8 (14.5%)	
Scores	Death (N=78)	Discharged (N=55)	p-Value
NEWS2*	6.63 (2.82)	5.88 (2.56)	0.148
MEWS*	3.51 (2.12)	3.61 (2.03)	0.81
qCSI*	3.93 (2.60)	4.38 (2.36)	0.324
4C*	11.5 (3.70)	9.35 (3.55)	0.002
VACO*	10.8 (10.6)	5.80 (7.67)	0.002
MulBTSA*	9.90 (2.62)	9.22 (3.30)	0.207
PRIEST COVID-19 clinical severity score*	10.7 (3.57)	9.10 (3.16)	0.012

Among the patients who arrived at the ICU on MV, more patients died (69.2%) than were discharged (14.5%; p <0.001). Furthermore, among the patients who died, 69.2% had arrived on MV while those discharged most (85.5%) had arrived at ICU on non-invasive ventilation (NIV). In the mortality group, all patients eventually required MV at some stage during their ICU stay (p<0.001). On average, the patients who died were intubated between day 2 and day 3 in ICU, while discharged patients were intubated on average within the first ICU day.

Most of the patients in both groups had not received antiviral therapy (Oseltamivir ± Kaletra). Plasma exchange was performed for 20.5% of the patients who died, and for 7.27% of those discharged. Out of the 78 patients who died, 32 (41.1%) received Dexamethasone. Anakinra was used only for 3.85% of the patients who died, and for 5.45% of the discharged patients. Intravenous immunoglobulin (IVIG) was used in a minority of patients who died (2.56%), and less frequently among discharged patients (1.82%). Among the patients who died, 46.2% needed proning, while among the discharged patients 30.9% required proning. In-hospital complications developed in most of the patients who died (94.9%), compared to 54.5% of those who were discharged (p<0.001). The most prominent of which were AKI, cardiac arrest, coagulopathy, and septic shock.

The average MEWS score was similar in the two groups of patients, however, the average NEWS2 severity score, qCSI score, and MulBTSA score were all on average higher in patients who died than those who were discharged (Table 10). The 4C and VACO scores were on average higher for the patients who died than those for discharged patients (p=0.002). While the average PRIEST COVID-19 clinical severity score was also higher amongst those who died (p 0.012).

**Table 10:** Early warning scores 7n (%); median (IQR). MEWS: Modified Early Warning Score for Clinical Deterioration, NEWS2: National Early Warning Score 2, qCSI: Quick COVID-19 Severity Index, 4C score: International Severe Acute Respiratory Infection Consortium Clinical Characterization Protocol (4C mortality score for COVID-19), VACO Index: Veterans Health Administration COVID-19 Index for COVID-19 Mortality, MuLBSTA: MuLBSTA Score for Viral Pneumonia Mortality, PRIEST score: Pandemic Respiratory Infection Emergency System Triage Severity Score.

Scores	N = 1 33^7^
NEWS2	6.00 (5.00, 8.00)
Unknown	19
MEWS	3.00 (2.00, 5.00)
Unknown	17
qCSI	5 (2.00, 7.00)
Unknown	12
4C	11.0 (8.0, 13.0)
Unknown	17
VACO (%)	5 (0, 12)
MulBTSA	9.00 (7.00, 12.00)
PRIEST COVID-19 clinical severity score	10.0 (8.0, 12.0)
Unknown	13

**Figure 1:**
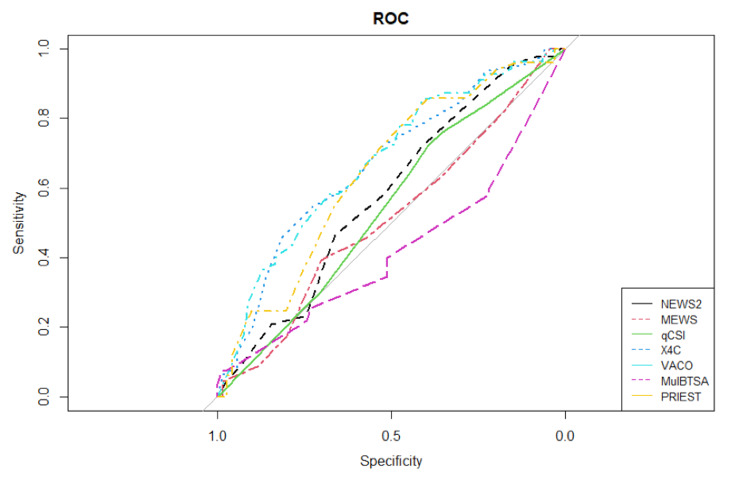
Area under the receiver operating characteristics graphs for each score

On average the COVID-19 scores were higher in the non-survivor group, as shown in Table 11. The high values of sensitivity (true positive rate), specificity (true negative rate), and AUC metrics, calculated for each score, show that all scores have a contribution to the prediction of in-hospital COVID-19 mortality. The AUROC graphs for each score are displayed in Figure 1.

**Table 11:** Early warning scores *Mean (SD). MEWS: Modified Early Warning Score for Clinical Deterioration, NEWS2: National Early Warning Score 2, qCSI: Quick COVID-19 Severity Index, 4C score: International Severe Acute Respiratory Infection Consortium Clinical Characterization Protocol (4C mortality score for COVID-19), VACO Index: Veterans Health Administration COVID-19 Index for COVID-19 Mortality, MuLBSTA: MuLBSTA Score for Viral Pneumonia Mortality, PRIEST score: Pandemic Respiratory Infection Emergency System Triage Severity Score.

COVID-19 scores	All cases (n = 133)	Death (N=78)	Discharged (N=55)	Cut-off	Sensitivity	Specificity	AUC (95% CI)
NEWS2*	6.35 (2.74)	6.63 (2.82)	5.88 (2.56)	7.5	0.7209	0.4085	0.5780 (0.4721–0.6838)
MEWS*	3.55 (2.07)	3.51 (2.12)	3.61 (2.03)	4.5	0.3913	0.7000	0.5124 (0.4051–0.6197)
qCSI*	4.12 (2.50)	3.93 (2.60)	4.38 (2.36)	3.5	0.7200	0.3944	0.5423 (0.4409–0.6436)
4C*	10.67 (3.78)	11.5 (3.70)	9.35 (3.55)	9.5	0.5435	0.7286	0.6682 (0.5675–0.7688)
VACO*	8.72 (9.75)	10.8 (10.6)	5.80 (7.67)	11.3	0.8545	0.4103	0.6761 (0.5837–0.7686)
MuIBTSA*	9.62 (2.93)	9.90 (2.62)	9.22 (3.30)	14.5	0.0727	0.9872	0.4214 (0.3206–0.5223)
PRIEST*	10.03 (3.49)	10.7 (3.57)	9.10 (3.16)	11.5	0.8571	0.3944	0.6452 (0.5459–0.7444)

Table 12 shows statistically significant associations between in-hospital mortality from COVID-19 and age (OR=0.95), direct admission to ICU from ED (OR=2.57), taking ACEI (OR=2.57), having CKD (OR = 0.25), WBC (OR=0.94), LDH (OR= 1.00), lactate (OR=0.79), urea (OR=0.94), albumin (0.004) (OR=1.10), arrival to ICU on MV (OR=12.8), delay in intubation (OR=0.69), duration of MV (OR=0.91), Dexamethasone (OR=2.31), developing in-hospital complications (OR=0.07), in-hospital AKI (OR=0.20), 4C mortality score for COVID-19 (OR=0.85), VACO Index for COVID-19 mortality (OR=0.93), and PRIEST COVID-19 clinical severity score (OR=0.87).

**Table 12:** Odds ratios of significant variables with 95% confidence interval and p-values

	Death (N=78)	Discharged (N=55)	OR	p-Value (OR)	p-Value (overall)
Age	62.6 (13.7)	53.2 (14.5)	0.95 [0.93;0.98]	0.001	<0.001
Direct admission to ICU					0.016
No (ward first)	58 (74.4%)	29 (52.7%)	Ref.	Ref.	
Yes (from ED to ICU)	20 (25.6%)	26 (47.3%)	2.57 [1.24;5.45]	0.011	
Taking ACEI					0.026
No	63 (80.8%)	34 (61.8%)	Ref.	Ref.	
Yes	15 (19.2%)	21 (38.2%)	2.57 [1.18;5.74]	0.018	
CKD					0.02
No	59 (75.6%)	51 (92.7%)	Ref.	Ref.	
Yes	19 (24.4%)	4 (7.27%)	0.25 [0.07;0.73]	0.01	
DBP	71.8 (14.4)	78.4 (12.7)	1.04 [1.01;1.07]	0.015	0.009
WBC	15.1 (11.8)	10.9 (5.35)	0.94 [0.89;0.99]	0.017	0.007
LDH	745 (348)	515 (209)	1.00 [1.00;1.00]	<0.001	<0.001
Lactate	3.92 (3.82)	2.20 (1.77)	0.79 [0.64;0.96]	0.02	0.003
Urea	18.3 (13.5)	11.7 (10.0)	0.94 [0.91;0.98]	0.005	0.002
Albumin	29.1 (6.48)	31.9 (4.63)	1.10 [1.03;1.18]	0.007	0.004
Arrival at ICU on					<0.001
MV	54 (69.2%)	8 (14.5%)	Ref.	Ref.	
NIV	24 (30.8%)	47 (85.5%)	12.8 [5.45;33.3]	<0.001	
Intubated on ICU day	2.50 (3.02)	0.96 (1.82)	0.69 [0.54;0.90]	0.005	<0.001
Duration of mechanical ventilation (days)	7.03 (6.52)	3.51 (6.59)	0.91 [0.85;0.97]	0.005	0.003
Dexamethasone					0.029
No	46 (59.0%)	21 (38.2%)	Ref.	Ref.	
Yes	32 (41.0%)	34 (61.8%)	2.31 [1.14;4.75]	0.02	
In-hospital complications					<0.001
No	4 (5.13%)	25 (45.5%)	Ref.	Ref.	
Yes	74 (94.9%)	30 (54.5%)	0.07 [0.02;0.20]	<0.001	
In-hospital AKI					<0.001
No	30 (38.5%)	42 (76.4%)	Ref.	Ref.	
Yes	48 (61.5%)	13 (23.6%)	0.20 [0.09;0.42]	<0.001	
VACO	10.8 (10.6)	5.80 (7.67)	0.93 [0.89;0.98]	0.006	0.002
4C	11.5 (3.70)	9.35 (3.55)	0.85 [0.76;0.95]	0.003	0.002
PRIEST COVID19 clinical severity score	10.7 (3.57)	9.10 (3.16)	0.87 [0.78;0.98]	0.018	0.012

A multivariable logistic regression model was built, including the variables that showed statistically significant association with in-hospital mortality. Arrival to ICU on MV (OR=45.76), delay in intubation (OR=0.51), developing in-hospital complications (OR=0.1) and having CKD (0.21) remained significant, as shown in Table 13.

**Table 13:** Multivariable analysis of clinical characteristics influencing COVID-19 mortality

Variable	p-Value	OR	C.I. (95%)
Arrival at ICU on MV	<0.001	45.76	(13.18, 204.71)
Day of intubation in ICU	0.0006	0.51	(0.33, 0.71)
In-hospital complications	0.006	0.1	(0.02, 0.49)
Having CKD	0.07	0.21	(0.03, 1.02)

## Discussion

Based on our study of 133 patients admitted to the COVID-19 ICU, demographically the majority of the patients were older males, with underlying comorbidities, most commonly diabetes mellitus and hypertension. The majority developed in-hospital complications, namely AKI, cardiac arrest with the return of spontaneous circulation, and coagulopathy, and the rates of which were all higher in the mortality group. Risk factors that were statistically significant as determinants of in-hospital mortality were found to be older age, delayed ICU care, and previously having CKD. On the other hand, laboratory findings that were elevated in the mortality group were LDH, lactate, urea, WBC, and albumin. It was observed that better outcomes were seen in those with early intubation during their ICU stay.

In our study, younger patients developed more favorable outcomes as compared to their older counterparts. The death rate was also noticed to be higher amongst males and those with co-morbidities. This is supported by a systematic review by Mehraeen et al. whereby older age and co-morbidities were found to be strong predictors of in-hospital admission and most importantly critical illness in patients with COVID-19 [20]. In a meta-analysis involving 18,012 patients and a systematic review of 15,794 patients, the most common comorbidities amongst critically ill COVID-19 patients was found to be diabetes and hypertension [21,22]. This paralleled with our results whereby those conditions predominated in our cohort.

However, there seems to be a debate on whether diabetes alone or synergistically with hypertension predicts severity and in-hospital mortality in those affected by the virus. Interestingly Sun et al. concluded that diabetes and raised blood glucose level, not a raised arterial blood pressure, is an independent predictor for the development of acute respiratory distress syndrome (ARDS) and subsequent respiratory failure in COVID-19 patients [23,24]. Nonetheless, the presence of co-morbidities in those affected by the virus equates to a higher likelihood of critical illness.

Extensive research has been undertaken to study the virulence of the SARS-CoV-2 virus, and it has been hypothesized that the virus gains entry into the host cells by binding to the angiotensin-converting enzyme 2 (ACE-2) receptors [24,25]. Such receptors are expressed by epithelial cells of the lungs, intestines, kidneys, brain, and blood vessels [24,25]. Animal studies have insinuated that ACEI and angiotensin-receptor blockers (ARBs) may increase the expression of ACE2 receptors [26]. This upregulation of the ACE2 receptor could potentially facilitate viral entry, providing an explanation for the increased severity of infection in those on ACEI or ARBs [24,25]. This can partly explain the high prevalence of diabetes and hypertension amongst critically ill COVID-19 patients since the majority of such patients are prescribed ACEI or ARBs [27,28].

In contradiction to the aforementioned theory, a number of studies including ours, report findings of improved outcomes in patients being treated with the above medications. A multi-center study by Fang et al. revealed a lower risk of all-cause in-hospital mortality among hypertensive COVID-19 patients being treated with ACE-I or ARBs [28].

On the other hand, in Brazil, a randomized control trial (RCT) of 659 patients hospitalized with a mild to moderate COVID-19 infection who were on a prescription of ACEIs or ARBs prior to hospitalization, investigated the effect of continuing versus discontinuing ACEIs or ARBs during the hospital stay. No difference in outcome was observed when comparing both groups [29]. The lack of consistency in the current literature is apparent, nevertheless, it can be safe to say that there is no role in discontinuing ACEIs or ARBs among patients hospitalized with COVID-19 provided there is an indication for treatment.

In our cohort, the presence of CKD rendered the patients more susceptible to in-hospital mortality. It is well established that CKD is an independent risk factor for severe COVID-19 disease and mortality, after adjusting for other concurring comorbidities [30]. In a meta-analysis involving 1389 patients, those with a previous history of CKD are at a threefold increased risk of developing severe COVID-19 disease [31]. Furthermore, it is in the etiological nature of CKD that such patients will also suffer from multiple concomitant comorbidities. The uremia-induced immune dysfunction associated with CKD has been suggested to play a role in the severity of infections in such patients [32]. It is safe to say that the presence of CKD as a comorbidity is a significant poor prognostic factor in COVID-19 patients, in addition to being a reason for prioritizing these patients for vaccination.

The development of in-hospital complications was observed more commonly in those who died, with the most significant being AKI, cardiac arrest with ROSC, coagulopathy, and septic shock. These predominated amongst the deceased group as opposed to those discharged and were similarly witnessed in multiple studies [33,34].

We observed other factors that increase the probability of in-hospital mortality, including delays in ICU admission and delays in intubation. Optimal timing for intubation and the definition of "early" intubation in patients with severe acute hypoxemic respiratory failure remains a controversial topic. It is no doubt that MV is associated with its own immediate and delayed complications, however, deferred intubation may also have detrimental consequences. Generally speaking in critically ill patients requiring intubation, it was previously established that a two-day delay in intubation is associated with increased mortality during the hospital stay [35].

This was supported more recently in a multi-center study conducted in New York specifically on COVID-19 patients. Similar to our findings, the study showed that in patients requiring MV for severe COVID-19, delays in intubation after admission was associated with higher mortality [36]. This is a crucial point to keep in mind during counseling patient’s relatives and clinical decision-making.

On the other hand, Papousti et al. conducted a systematic review and a meta-analysis, whereby “early” intubation was defined as intubation within 24 hours from admission. Findings were reported showing no statistically detectable difference in all-cause mortality, duration of MV, and ICU length of stay between patients undergoing early versus late intubation [37].

Fundamentally, decisions on the timing of intubation should rely on the clinical judgment of experienced intensivists while keeping in mind the oxygen status and the level of respiratory distress the patient is experiencing. In those requiring higher oxygen support, our center prefers a trial of high flow nasal oxygen (HFNO) or NIV rather than proceeding directly to intubation, after risk/benefit evaluation and taking into consideration efficient resource allocation [38].

Steroid therapy has shown to be the sole intervention to significantly reduce mortality in patients with COVID-19. As reported by the RECOVERY TRIAL, dexamethasone specifically has proven its efficacy in reducing mortality when compared to conventional therapy [39]. It has been postulated that glucocorticoids down-regulate the pathogenesis of the virus and its subsequent complications by means of modulating the host inflammatory pathways, reducing vascular and endothelial wall inflammation, and in turn reducing organ and tissue injury, edema formation, and the risk of arterial and venous occlusion [40]. Similarly, it was noticed in our study that more patients amongst the survival group were prescribed dexamethasone than those in the mortality group.

Many prognostic and early warning scores have been utilized in COVID-19 patients. They each vary in the clinical parameters included and ease of applicability, however, the ultimate goal of such scoring systems is to predict patient prognosis and/or in-hospital mortality. This serves as an adjunct to ease clinical decision-making, triaging, resource allocation, and/or patient counseling. For each of our patients, we calculated seven early warning scores that have been previously used in COVID-19 patients, and we examined their performance in predicting in-hospital mortality in our patient cohort. To the best of our knowledge, our study is one of few to compare seven early warning scores in COVID-19 patients admitted to the ICU.

The scores studied were the MEWS for clinical deterioration [12], the NEWS2 score [13], qCSI [14], the International Severe Acute Respiratory Infection Consortium Clinical Characterization Protocol (4C mortality score for COVID-19) [15], the VACO index for COVID-19 mortality [16], the MuLBSTA Score for Viral Pneumonia Mortality (MulBTSA) [17], and the PRIEST [18]. All scores were observed to have the ability to predict in-hospital mortality, however, the 4C, VACO, and the PRIEST severity scores yielded significant findings compared to the remaining four scores. The NEWS, MEWS, qCSI scores mainly focused on vital sign stability, i.e., respiratory rate, saturation, temperature, pulse, and blood pressure. Whereas, the 4C, VACO, and PRIEST scores had a wider range of variables, especially the inclusion of comorbidities, gender, and age, the importance of which has been aforementioned and discussed as strong predictors of severity and mortality, which allowed for a more comprehensive assessment of each patient [41].

The 4C, VACO, and PRIEST scores proved to be the three most superior scores, having the highest AUC (area under the ROC Curve) values. The VACO and the PRIEST scores had the highest sensitivity of all the seven examined scores, while the sensitivity of the 4C score was satisfactory. However, the specificity of the 4C score was exceedingly higher than that of the VACO and PRIEST scores. When comparing the three most accurate scores, the 4C score proved to be less sensitive yet the most specific when compared to the VACO and PRIEST scores (Table 11).

Limitations

Our study is accompanied by a few limitations. First, the limited sample size, our study is a single-center experience and due to the lack of an electronic-based system linking our COVID-19 centers, a multi-center study was not undertaken. In addition, certain clinical parameters that could potentially influence the outcome of COVID-19 patients were not included, for example, chest X-ray findings, arterial blood gas analysis, patient weight/body mass index, and ventilator parameters. Also, due to the nature of the study, we could not assess the long-term complications and/or death rate in those who were discharged from the ICU.

## Conclusions

The early identification of those likely to have an unfavorable prognosis allows for efficient triaging, adequate resource allocation, and the delivery of a high standard of care. In our experience certain demographic and clinical parameters were identified as early predictors of an adverse outcome; furthermore, the use of early warning scores can aid in clinical decision-making. In our experience, the 4C mortality score for COVID-19, the VACO index for COVID-19 mortality, and the PRIEST COVID-19 clinical severity score proved to be superior compared to the rest of the scores, leaving the choice down to personal preference and convenience in applicability.
